# Educational technology to promote self-efficacy in newborn care: a validation study

**DOI:** 10.17533/udea.iee.v42n2e14

**Published:** 2024-07-09

**Authors:** Jallyne Colares Bezerra, Hévila Ferreira Gomes Medeiros Braga, Antônio Marcos de Souza Soares, Maria Jocelane Nascimento da Silva, Antônia Ellen Jardani de Souza Medeiros, Emília Soares Chaves Rouberte, Flávia Paula Magalhães Monteiro, Emanuella Silva Joventino Melo

**Affiliations:** 1 Nurse, Master. Nurse at Fortaleza General Hospital, Fortaleza, Ceará, Brazil. Email: jallynecolares@gmail.com Hospital Geral de Fortaleza Fortaleza General Hospital Fortaleza Ceará Brazil jallynecolares@gmail.com; 2 Nurse, Master Student. Email: hevila.medeiros.hm@gmail.com. Corresponding author. Universidade da Integração Internacional da Lusofonia Afro-Brasileira Brazil hevila.medeiros.hm@gmail.com; 3 Graduation Student. Email: marcossouza@aluno.unilab.edu.br Universidade da Integração Internacional da Lusofonia Afro-Brasileira Brazil marcossouza@aluno.unilab.edu.br; 4 Nurse, Master. Email: jocelane.nascimento.silva@gmail.com Universidade da Integração Internacional da Lusofonia Afro-Brasileira Brazil jocelane.nascimento.silva@gmail.com; 5 Nurse. Nurse at Doctor Dilberto Prata Mota Health Center, Redenção, Ceará, Brazil. Email: jardanimedeiros@gmail.com Dilberto Prata Mota Health Center Doctor Dilberto Prata Mota Health Center Redenção Ceará Brazil jardanimedeiros@gmail.com; 6 Nurse, Ph.D. Full Professor. Email: emilia@unilab.edu.br Universidade da Integração Internacional da Lusofonia Afro-Brasileira Brazil emilia@unilab.edu.br; 7 Nurse, Ph. D. Full Professor. Email: flaviapmm@unilab.edu.br Universidade da Integração Internacional da Lusofonia Afro-Brasileira Brazil flaviapmm@unilab.edu.br; 8 Nurse, Ph. D. Full Professor. Email: ejoventino@unilab.edu.br Universidade da Integração Internacional da Lusofonia Afro-Brasileira Brazil ejoventino@unilab.edu.br; 9 University of International Integration of Afro-Brazilian Lusophony, Redenção, Ceará, Brazil. Universidade da Integração Internacional da Lusofonia Afro-Brasileira University of International Integration of Afro-Brazilian Lusophony Redenção Ceará Brazil

**Keywords:** educational technology, validation study, newborn, health education, nursing, tecnologia educacional, estudios de validación, recién nacido, educación para la salud, enfermería., tecnologia educacional, estudos de validação, recém-nascido, educação em saúde, enfermagem.

## Abstract

**Objective.:**

To build and validate an educational technology consisting of a flipchart to promote self-efficacy in newborn care.

**Methods.:**

A methodological study was carried out in two stages: (i) creation of the flipchart and (ii) validation by 25 experts and 50 people who could be the target audience (pregnant women, mothers or family members of newborns). Clarity, language, practical relevance and theoretical relevance were reviewed using the Suitability Assessment of Materials (SAM) instrument. The Content Validity Index and the Flesch Readability Index were calculated.

**Results.:**

The serial album "Can you take care of your baby" consists of 30 pages. The overall Content Validity Index was 0.93 among experts and 1.0 among the target audience. The flipchart was considered superior quality material, reaching an agreement percentage of 94.9, indicating that it is suitable as an educational technology. Participants suggested adjustments, incorporated into the material for printed production.

**Conclusions.:**

The flipchart developed and with content validated by experts is suitable for use in health education activities that aim to promote self-efficacy in caring for newborns.

## Resumen

**Objetivo.:**

Construir y validar una tecnología educativa consistente en un rotafolio para promover la autoeficacia en el cuidado del recién nacido.

**Métodos.:**

Se realizó un estudio metodológico en dos etapas: (i) creación del rotafolio y (ii) validación por parte de 25 expertos y 50 personas que podrían ser el público objetivo (mujeres embarazadas, madres o familiares de recién nacidos). La claridad, el lenguaje, la relevancia práctica y la relevancia teórica se revisaron utilizando el instrumento de Evaluación de idoneidad de materiales (SAM). Se calcularon el Índice de Validez de Contenido y el Índice de Legibilidad de Flesch.

**Resultados.:**

El rotafolio "¿Puedes cuidar a tu bebé?" tuvo un Índice de Validez de Contenido general de 0.93 entre los expertos y de 1.0 entre el público objetivo. Este rotafolio fue considerado como material educativo de calidad superior, alcanzando un porcentaje de acuerdo de 94.9%, indicando que es apto como tecnología educativa. Los participantes sugirieron ajustes que fueron, incorporados al material para la producción impresa.

**Conclusiones.:**

El rotafolio desarrollado y con contenido validado por expertos es apto para su uso en actividades de educación en salud que tengan como objetivo promover la autoeficacia en el cuidado del recién nacido.

## Resumo

**Introdução.:**

Construir e validar uma tecnologia educativa composta por álbum seriado para promover a autoeficácia no cuidado ao recém-nascido.

**Métodos.:**

Foi realizado um estudo metodológico em duas etapas: (i) criação do álbum seriado e (ii) validação por 25 especialistas e 50 pessoas que poderiam ser o público-alvo (gestantes, mães ou familiares de recém-nascidos). A clareza, a linguagem, a relevância prática e a relevância teórica foram revisadas por meio do instrumento Suitability Assessment of Materials (SAM). Foram calculados o Índice de Validade de Conteúdo e o Índice de Legibilidade de Flesch.

**Resultados.:**

O álbum seriado "Você consegue cuidar do seu bebê" consiste em 30 páginas. O Índice de Validade de Conteúdo geral foi de 0.93 entre os especialistas e de 1.0 entre o público-alvo. O álbum seriado foi considerado um material de qualidade superior, atingindo percentual de concordância de 94.9, indicando que é adequado como tecnologia educativa. Os participantes sugeriram ajustes, incorporados ao material para a produção impressa.

**Conclusões.:**

O álbum seriado desenvolvido e com conteúdo validado por especialistas é adequado para utilização em atividades de educação em saúde que visam promover a autoeficácia no cuidado ao recém-nascido.

## Introduction

In 2022, the neonatal mortality rate in Brazil reached the mark of 8600 live births. Despite this, there are inequalities in the trends of preventable neonatal mortality rates in the Brazilian states, so they remain higher in the North and Northeast regions, demonstrating the need to improve access and quality of maternal and child health care.^1^ Educational interventions prioritizing improvements in care for the neonatal public, such as printed educational technologies, are necessary. The development of this type of technology allows contacting the patient dynamically and attractively, capable of awakening the patient's attention to important situations, stimulating discussions, resolving doubts, recognizing risks, and establishing achievable goals.^2^

Album-type printed technologies developed with methodological rigor, and well-formulated content can raise user awareness, encourage self-care, and improve the quality of clinical care, especially in primary health care.^3^^,^^4^ A participatory, communicative, and collective approach is necessary for creating any educational material.^5^ Thus, this type of technology allows for greater integrity between the educator and learner and can enhance health education carried out by professionals.^6^ Health professionals who use this type of technology can direct the sequence of exposition of the script, resume any information already presented, and mark the most important points in the script sheets. Besides, flipcharts are a portable alternative that can be used in different healthcare settings.^6^

In the context of newborn care, mothers, fathers, and family members generally feel insecure and unprepared when the newborn arrives home.^7^ To this end, incorporating the concept of self-efficacy in educational interventions and technologies raises the individuals' beliefs about their abilities to successfully carry out the intended actions.^8^ High levels of self-efficacy can help in promoting health and therapeutic adherence, leading to better clinical results, reducing processes of exacerbation or aggravation, and demonstrating benefits in the short, medium, and long term for the patient, the family, and the health system, even in the face of adverse conditions.^9^ As a technology applied by health professionals, the flipchart encourages interaction between the professional and the target audience, helping to exchange knowledge and strengthen relationships. For reliable use, such educational materials must be submitted to a rigorous validation process by researchers, health professionals, and the target audience. The objective of this study was to build and validate an educational technology consisting of a flipchart to promote self-efficacy in newborn care. 

## Methods

A methodological study was conducted in two stages: (i) the creation of the flipchart and (ii) content and appearance validation by experts and target audiences. The construction and validation stage took place from October 2020 to February 2022, in municipalities of the state of Ceará, Brazil. The study was described according to the SQUIRE 2.0 (Equator Network) guidelines.Once constructed, the flipchart was validated by a committee composed of an expert in neonatal health, child health, family/community/public health, and technical experts. All experts met at least two requirements: having knowledge/skills acquired through experience and special knowledge/skills in a certain type of study on the subject.^10^

The sample size was defined using a formula that considers the final proportion of experts concerning a dichotomous variable and the maximum acceptable difference in this proportion: *n* = Zα².P.(1-P)/d², where Zα refers to the confidence level (95%), P is the minimum proportion of individuals who agree with the pertinence of the items (85%), and d is the difference in proportion considered acceptable (5%).^11^ The final calculation resulted in a sample of 22 experts; however, it is noteworthy that an odd number of experts is essential to avoid ties.^12^ A larger number of experts was recruited to avoid losses, and 25 experts participated in the study and returned the instruments.

The validation step was conducted with a convenience sample whose size respected the recommendation of recruiting 25 to 50 subjects to validate technologies/instruments.^13^ Thus, 50 pregnant women, mothers, and family members participated. The following inclusion criteria were adopted: being a pregnant woman, a recent mother or a father of a child aged up to 28 days, or a close relative (such as the child's grandmother or aunt/uncle). Participants with difficulty understanding were excluded, as pregnant women, mothers, and relatives of premature newborns, since the flipchart addresses caring routines for full-term newborns.

During the development of the flipchart, a literature review was conducted in October 2020 in Cumulative Index to Nursing and Allied Health Literature (CINAHL), US National Library of Medicine (PubMed), Web of Science, and Latin American and Caribbean Literature in Health Sciences (LILACS) databases. The following guiding question was established: What are the main nursing practices in newborn health care? For this purpose, the descriptors "newborn", "health education", and "nursing" were used combined by the Boolean operator "AND". Thirty-eight studies were selected that addressed themes related to the care provided to the newborn, namely: breastfeeding, sleep, colic management, pain control, sunbathing, immunization, neonatal screening, umbilical stump care, hygiene, interpersonal bonding, and identification of alarm signals.

A professional designer was hired to elaborate the flipchart's figures and layout using the Adobe InDesign image editing software. As the illustrations were initially made in paper drawings, the researchers approved or suggested changes to improve clarity and representativeness so that the illustrator could transfer the figures to Adobe InDesign. It should be noted that the flipchart was built based on validated technologies, namely: the Self-efficacy Scale for the Promotion of Care to Term Newborns (SSPCTN)^14^ and the educational video entitled "Taking care of your baby".^15^

The recruitment of experts for the content validation stage was done through the Lattes Platform of the Brazilian National Council for Scientific and Technological Development (CNPq). The experts who met the eligibility criteria received an invitation letter by e-mail explaining the purpose of the research, together with a request to indicate other specialists who met the selection criteria (snowball technique). Thirty-nine experts were invited, 25 returned the e-mail, 11 were teaching experts, 11 were assistant experts, and 3 were technical experts. Data collection took place using the Google Forms tool, via e-mail, with the following documents: ICF, a questionnaire for the characterization of the experts, including academic and professional data, an instrument assessing the appearance and content of figures and script sheets, and the first version of the flipchart.

The validation instrument was a Likert-type scale with five points, ranging from "poor" to "excellent", allowing the assessment of the images and script sheets based on the following criteria: clarity of language, practical relevance, and theoretical relevance. In addition, there was a space for the experts' suggestions. The instrument used for the evaluation was the Suitability Assessment of Materials (SAM), which contained variables on the following domains: content, appropriate language for the population, graphic illustrations, lists, tables and graphs, layout and typography, stimulation for learning, and motivation, and cultural adequacy.^16^ SAM scores are evaluated as “superior”, meaning 2 points; “adequate” 1 point; and “inadequate”, 0 points, according to objective criteria included in the instrument that enable both the calculation of the average values and the analysis percentage.The SAM questionnaire rates "superior" materials as those that reach a final score of 70% to 100%. Scores from 40% to 69% indicate an "adequate" material, and scores from 0% to 39% indicate an "inadequate" material.^16^

An analysis was performed using Microsoft OfficeWord version 2010 to the interpretation of the values ​​obtained with the Readability Index, considering the following assumptions: 75 - 100% (very easy), 50 - 75% (easy), 25 - 50% (difficult), and 0 - 25% (very difficult).^17^ The participants' responses regarding clarity and relevance were used for appearance validation, considering the cut-off agreement of 75%.^18^ The deadline for returning the instruments was 30 days.

After validation and evaluation of the flipchart, all suggestions were analyzed, and a new contact was made with the technical professional responsible for the illustration and layout so that modifications and adjustments could be made according to the experts' recommendations. In addition, some texts were modified according to the experts' suggestions, and then the Flesch Readability Index was applied to assess the text's reading level. This analysis considers that adequate instruments should present a value equal to or greater than 40% concerning the total scores.^17^

After improving the images and texts, the target audience individually validated and evaluated the flipchart. Data collection for this stage consisted of applying the ICF, applying the flipchart and, subsequently, answering a questionnaire consisting of four parts: 1) sociodemographic characterization; 2) evaluation of the domains of the adapted questionnaire by Doak, Doak, and Root (organization, understanding, attractiveness, self-efficacy, cultural acceptability, and persuasion);^16^ 3) Individual assessment of each image in the flipchart, using a checklist to assess clarity, relevance, and degree of relevance; and 4) Additional suggestions regarding the flipchart.

The data obtained were organized, processed, and analyzed using the Statistical Package for the Social Sciences (SPSS) version 20.0 and the R software. The Content Validity Index (CVI) was used to analyze the content validity. It is recommended that the CVI adopted as valid be equal to or greater than 0.80 and that values ​​greater than 0.90 ensure excellence in content validity.^19^ In the present study, an agreement of 80% was adopted among the participants.^20^The study was approved by the Research Ethics Committee (CAAE: 29622220.4.0000.5576). All research participants signed the Informed Consent Form, after being verbally informed about the objectives and procedures of the study.

## Results

The first version of the flipchart entitled "You are capable of taking care of your baby" contained 28 pages and was divided into 12 themes: (1) Sleep, (2) Bath, (3) Changing diapers, (4) Umbilical stump hygiene, (5) Cloth hygiene, (6) Immunization, (7) Sunbathing, (8) Breastfeeding, (9) Cramps, (10) Heel-stick test, (11) Warning signs, and (12) Choking. Regarding the content of the material, it was considered that the family context is usually composed of a mother, a father, a grandmother, and the newborn, in addition to the nurse, an important actor who helps with doubts related to the care of the newborn. It was decided to include other family members, such as the child's father and grandmother, to show that care should and can be performed by all family members, not just the mother.

In addition, we sought to use simple and direct language in the care guidelines described in each script sheet, bringing the illustrations closer to the cultural reality of the target audience. Thus, based on the subject description, the readability test was applied in 39 (100%) paragraphs/sentences from the flipchart. Of these, 11 (28.2%) were considered "very easy", 20 (51.3%) were considered "easy", 6 (15.4%) were considered "difficult," and 3 (7.7%) were considered "very difficult". Regarding the classification by theme, of the 12 themes, 10 obtained an "easy" classification, and 2 obtained a "difficult" classification, namely, '9/colic' and '11/warning signs'. In the analysis of the complete flipchart, the test revealed an index of 60.4%, classifying the material as easy to read and understand.

The first version was validated by 25 experts divided into 3 categories: 11 professors, 11 assistants, and 3 technicians. All professor experts were female, with academic training in nursing (90.9%), specialization training (45.5%), master's degree (90.1%), or doctoral degree (81.8%). Concerning care experts, all were female, with academic training in nursing (90.9%), and two had completed specializations training (78.2%). As for the technical experts, 66.7% were female, with an average of four years of experience producing educational materials. The CVI of each page was calculated considering the clarity of the language, the practical pertinence, and theoretical relevance, and, subsequently, the overall CVI (Table 1).

**Table 1:** Distribution of the CVI of each page, according to the analysis of the content experts

Page/Subject	Clarity of language	Practical relevance	Theoretical relevance
Front cover	0.90	0.90	0.91
Page 5/ Sleep	0.90	0.94	0.94
Page 7 Bath	0.86	0.90	0.92
Page 9/ Changing diapers	0.92	0.96	0.96
Page 11/ Umbilical stump hygiene	0.93	0.98	0.95
Page 13/ Cloth hygiene	0.93	0.91	0.92
Page 15/ Immunization	0.84	0.96	0.93
Page 17/ Sunbathing	0.92	0.94	0.93
Page 19/ Breastfeeding	0.95	0.95	0.97
Page 21/ Cramps	0.92	0.92	0.93
Page 23/ Heel-stick test	0.95	0.95	0.94
Page 25/ Warning signs	0.90	0.91	0.92
Page 27/ Choking	0.90	0.96	0.97
Global CVI	0.91	0.94	0.94

As for language clarity, all pages had a CVI greater than or equal to 0.84. Concerning practical pertinence and theoretical relevance, all pages had a CVI greater than or equal to 0.90. The three categories obtained a total above 0.90, and the global CVI was 0.93, indicating an excellent level of approval and agreement. The experts also evaluated the flipchart using the SAM instrument. The flipchart was considered a superior quality material, reaching a percentage of agreement of 94.9. (Table 2).

**Table 2:** Flipchart evaluation by content and technical of 25 experts

Domains	Superior *n* (%)	Adequate *n* (%)	Inadequate *n* (%)	Total Agreement (%)
Content				
The purpose is evident	25 (100)	0	0	100
The content addresses behaviors	24 (96)	1 (4)	0	98
The proposal is limited	24 (96)	1 (4)	0	98
Summary or review	22 (88)	3 (12)	0	94
Appropriate language for the population				
Reading level	15 (60)	7 (28)	3 (12)	74
Active voice style	16 (54)	8 (32)	1 (4)	80
Use of common words	21 (84)	3 (12)	1 (4)	90
First, the context	23 (92)	1 (4)	1 (3)	94
Advanced sign-mediated learning	24 (96)	1 (4)	0	98
Graphic illustrations, lists, and tables				
Front cover	22 (88)	3 (6)	0	94
Type of illustration	24 (96)	1 (4)	0	98
Relevance of lustrations	22 (88)	3 (12)	0	94
Lists, tables, charts, and shapes	25 (100)	0	0	
Captions	23 (92)	2 (8)	0	96
Layout and typography				
Layout factors	24 (96)	1 (4)	0	98
Typography	22 (88)	3 (12)	0	94
Subtitles	25 (100)	0	0	100
Stimulation for learning and motivation				
Interaction is included in the text and/or figures	24 (96)	1 (4)	0	98
Desired behavior patterns are modeled or shown through specific terms	24 (96)	1 (4)	0	98
Motivation/self-efficacy	25 (100)	0	0	100
Cultural adequacy				
Cultural game - logic, language, and experience (LLE)	24 (96)	1 (4)	0	98
Cultural image and examples	22 (88)	3 (12)	0	94
Total	500 (90.9)	44 (8)	6 (1.1)	94.9

The experts validated the flipchart, and with the adjustments already made, the second version of the flipchart was evaluated by the target audience represented by pregnant women, puerperal women, and family members attending primary healthcare centers in the municipalities of Redenção, Acarape, and Canindé. These participants had an average age of 27 years; most of them lived with a partner (70%) and had completed high school (68%). The target audience assessed the relevance of the appearance of the pictures on each page of the flipchart.The material presented a global CVI of 1.0, indicating a high level of agreement among the participants. In addition, the participants evaluated the domains of organization, understanding, attractiveness, and cultural acceptability positively and satisfactorily. All participants (*n*=50;100%) agreed that: (i) The cover presents the flipchart's subject and is attractive, (ii) The colors are adequate, and the figures help to understand the subject, and (iii) The flipchart addresses the actions to be conducted and feel like talking about the topic. Finally, the participants did not find any part of the material bad or aggressive.

**Figure 1:**
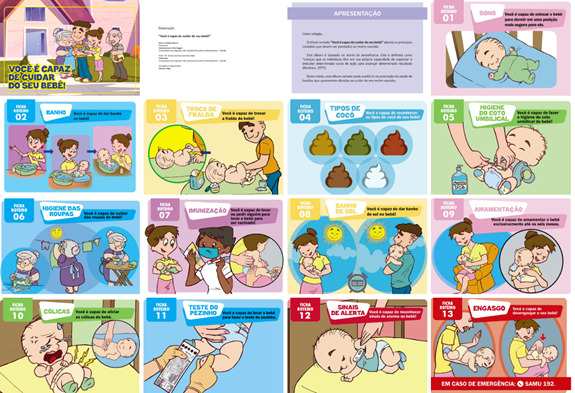
Illustrations from the flipchart "You are capable of taking care of your baby"

Most participants reported not knowing all the care actions necessary for newborn care (64%). However, all were predisposed and believed that they could follow the guidelines and would inform other people about the care for the newborn, as shown in the album, attesting to the power of self-efficacy and persuasiveness of the material. Finally, the target audience's suggestions were accepted, and the final version of the flipchart was created, including a picture and a script sheet on the aspects of the newborn's feces. Thus, the final version of the flipchart entitled "You are capable of taking care of your baby" consisted of 30 pages, including a cover, presentation, technical sheet (informing that the material was created by a master's student, a supervisor, and a graphic designer), 26 figures with the respective script sheets for the 13 subjects, and acknowledgments (Figure 1). The material is accessible through the following address: https://drive.google.com/file/d/1rUY_c7IQoZgvhvgjwmhzhlMgtYgYar1N/view?usp=sharing 

## Discussion

The validation of the flipchart's content resulted in a high CVI, with an excellent level of agreement among the experts, indicating that the flipchart is representative of the proposed content (newborn care). Validating educational technologies with experts is a relevant step, as the expertise of these professionals is taken into consideration through their opinions and comments, ensuring that the materials have adequate information.^21^ It is important to consider both the judgment of academic researchers and the expertise of professionals working in the healthcare field. Throughout the content validation, most experts judged the album's language and illustrations as appropriate and easy to understand. These two elements are fundamental to ensure that audiences with different levels of education will be able to understand and assimilate the content.^22^

Regarding the SAM score, it was observed that the flipchart achieved a score higher than the established cut-off, demonstrating the agreement of the experts. Although the material was well evaluated, the experts' observations and recommendations helped reformulate some textual information, modify illustrations, and revise and replace words. These changes were essential to improve the quality of the educational material. The scientific reliability of the information passed on is important. Besides, the constructed material must be easier for the target audience to understand.^23^

Validating the appearance of educational materials with individuals who experience or have experienced the theme addressed is an important activity since they are the focus of the activity to be conducted.^21^ In this study, the flipchart was positively evaluated by the target audience concerning clarity, language, and relevance, corroborating the advantages of using educational materials validated by the population, including enhanced interactivity, attractiveness, use of appropriate language, and provision of relevant and contextualized activities, allowing the exchange of experiences and presenting quality information.^24^ Given the above, there was a concern about making the suggested adjustments to achieve an understanding of the illustrations and the subjects addressed in the flipchart. One of the participants' suggestions was about diarrhea. Therefore, a figure and script sheet was added addressing the aspect of the feces of the newborn. The recommendation was considered as the presence of liquid stools several times a day in an infant can lead the mother or caregiver to think that he or she has diarrhea.The importance of using educational materials to prevent problems is evident since most participants reported not knowing all the precautions covered in the flipchart. There were reports that "drying clothes in the sun would cause illnesses", a lack of knowledge about the pathologies that the heel-stick identifies, and using teas with onions and herbs to clean the umbilical stump. Therefore, it is possible to verify that the flipchart has relevant content, easy language, and illustrations that facilitate understanding, promoting self-efficacy. Thus, it is hoped that after reading the flipchart, people will feel able to follow the information and provide care for their newborns.

No participant reported being offended or seeing anything aggressive in the album. In view of this, it is important to highlight the identification of facilitators and hindrances (social, cultural and epidemiological) so that educational strategies can be implemented aimed at the person's reality, with the outlining and reframing of new modes of care.^25^ Therefore, a technology such as a flipchart is a very interesting visual resource that can be used in different situations, such as health educational activities, respecting the cultural context in which the participants live.

Regarding the self-efficacy of the educational material, the participants stated that they intended to follow the guidelines presented in the album, and that if they had to inform another woman how to carry out the care, they would inform as shown. This confirms the fact that the serial album was constructed and applied following the four sources of self-efficacy, which play a role in the origin and development of self-efficacy beliefs, they are: experience of success; vicarious or modeling experience; verbal persuasion; and physiological states.^8^

In this sense, the serial album presents itself as a health promotion instrument that facilitates educational process. To this end, its purpose is to guide the dialogue between the health professional and individuals, as it allows cooperation between those involved in the knowledge construction process. Therefore, this resource, with its easy language and presence of images associated with the experience, provides an increase in understanding of the content presented.^22^ Furthermore, educational materials such as flipcharts must provide interactivity, be attractive, have language appropriate to the target audience, provide relevant and contextualized activities, allow the exchange of experiences and present quality information.^24^

As a conclusion of the study, the flipchart "You are capable of taking care of your baby" was considered adequate regarding content and appearance, promoting the self-efficacy of pregnant women, puerperal women, and family members for newborn care. The flipchart achieved a global CVI equal to 0.93 among the experts and 1.0 among the target audience. The flipchart was considered superior based on the final SAM score of 94.9%. The album is relevant as it is an educational resource for health education activities, providing clear information that ensures the assimilation of knowledge and decision-making by families about the most appropriate newborn care practices.

The flipchart can be used by health professionals, especially nurses carrying out educational interventions to promote self-efficacy in full-term newborn care. This educational technology is expected to be widely disseminated and used by nurses to empower the family for newborn care. As a limitation of the study, it is pointed out that the material was evaluated only by the target audience assisted in the public health network, which may differ from the reality of private institutions. However, it is believed that the flipchart will also be comprehensible for individuals with higher education, as it contains clear information and a good assessment of the degree of readability. In addition, the results may also differ from the reality of other Brazilian states.
